# Worldwide Occurrence of Integrative Conjugative Element Encoding Multidrug Resistance Determinants in Epidemic *Vibrio cholerae* O1

**DOI:** 10.1371/journal.pone.0108728

**Published:** 2014-09-29

**Authors:** Michel A. Marin, Erica L. Fonseca, Bruno N. Andrade, Adriana C. Cabral, Ana Carolina P. Vicente

**Affiliations:** Laboratory of Molecular Genetics of Microorganisms, Oswaldo Cruz Institute (IOC) - Oswaldo Cruz Foundation (FIOCRUZ), Rio de Janeiro, Brazil; Institut National de la Recherche Agronomique, France

## Abstract

In the last decades, there has been an increase of cholera epidemics caused by multidrug resistant strains. Particularly, the integrative and conjugative element (ICE) seems to play a major role in the emergence of multidrug resistant *Vibrio cholerae*. This study fully characterized, by whole genome sequencing, new ICEs carried by multidrug resistant *V. cholerae* O1 strains from Nigeria (2010) (ICE*Vch*Nig1) and Nepal (1994) (ICE*Vch*Nep1). The gene content and gene order of these two ICEs are the same, and identical to ICE*Vch*Ind5, ICE*Vch*Ban5 and ICE*Vch*Hai1 previously identified in multidrug resistant *V. cholerae* O1. This ICE is characterized by *dfrA1*, *sul2*, *strAB* and *floR* antimicrobial resistance genes, and by unique gene content in HS4 and HS5 ICE regions. Screening for ICEs, in publicly available *V. cholerae* genomes, revealed the occurrence and widespread distribution of this ICE among *V. cholerae* O1. Metagenomic analysis found segments of this ICE in marine environments far from the direct influence of the cholera epidemic. Therefore, this study revealed the epidemiology of a spatio-temporal prevalent ICE in *V. cholerae* O1. Its occurrence and dispersion in *V. cholerae* O1 strains from different continents throughout more than two decades can be indicative of its role in the fitness of the current pandemic lineage.

## Background

The burden of cholera has grown strikingly during the last years, and has spread to countries previously spared of this disease. An increasing number of multidrug resistant *Vibrio cholerae* strains have been reported in recent cholera outbreaks [1], and this resistance phenotype is mainly associated with the presence of mobile and mobilized elements, such as integrative and conjugative elements (ICEs). ICEs are self-transmissible mobile elements, able to integrate into the host bacterial chromosome, excise and transfer to a new host genome through conjugation [2]. To date, a dozen related ICEs belonging to the SXT/R391 family have been identified, which are characterized by a SXT integrase gene (*int*_SXT_) that enables site-specific integration of the ICE into the 5′ end of *prfC* gene. There are 52 core genes present in all SXT/R391 ICEs required for integration/excision, conjugative transfer, regulation, as well as many genes of unknown function. There are five regions considered hotspots (HS) for insertion of foreign DNA that afford specific characteristics to the bacterial host [3] [4] [5]. The HS content is variable but some ICEs occasionally share these contents. Additional variable DNA inserts (named variable regions) outside the HS are also present [5]. Differences in the HS and variable regions are responsible for ICE size variation, but mainly for specific traits, such as antimicrobial resistance [5] and fitness [6], that characterize the distinct SXT/R391 ICEs.

According to Mutreja et al [7], the 7^th^ cholera pandemic spread from the Bay of Bengal in three independent waves of global transmission, in which waves 2 and 3 were characterized by the acquisition of SXT/R391 ICEs.

The *V. cholerae* O1 lineage, responsible for the Haitian cholera epidemic in 2010, harbored an ICE (ICE*Vch*Hai1) characterized by the antimicrobial resistance genes *dfrA1* (trimethoprim), *sul2* (sulfamethoxazole), *strAB* (streptomycin) and *floR* (chloramphenicol) [8]. *V. cholerae* O1 strains harboring ICEs with this same set of antimicrobial resistance genes have also been reported in India (ICE*Vch*Ind5, 1994–2005) [9] [5] and Bangladesh (ICE*Vch*Ban5, 1998) [5].

Here, we fully characterized, by whole genome sequencing, new ICEs carried by multidrug resistant *V. cholerae* O1 strains from Nigeria (2010) (ICE*Vch*Nig1) and Nepal (1994) (ICE*Vch*Nep1). It was demonstrated that these two ICEs shared the same gene content and gene order with ICEs (ICE*Vch*Ind5, ICE*Vch*Ban5, ICE*Vch*Hai1) previously found in a set of multidrug resistant *V. cholerae* O1 strains isolated in different countries throughout more than two decades. These elements corresponded to a single ICE type, named here group 1, characterized by *dfrA1*, *sul2*, *strAB* and *floR* antimicrobial resistance genes, and by unique gene content in HS4 and HS5 regions. Segments of this ICE were found in marine metagenome datasets. Therefore, here, we compiled their genomic information, revealing the real scenario of this ICE group distribution, and proposed that HS4 and HS5 regions would be useful to trace the group 1 ICE.

## Results and Discussion

Using whole genome sequencing, ICEs were identified and characterized in multidrug-resistant *V. cholerae* O1 clinical strains from Nigeria (2010) and Nepal (1994). Surprisingly, comparing their entire genomic content and structure, we concluded that they are siblings of ICE*Vch*Hai1, ICE*Vch*Ind5 and ICE*Vch*Ban5, which were previously identified and characterized in multidrug-resistant strains causing outbreaks/epidemics in Haiti [8] [10], India [9] and Bangladesh [5], respectively. Moreover, searches in publicly available *V. cholerae* genomes revealed the widespread presence of this sibling ICE in strains from cholera outbreaks/epidemics in three continents over a period of more than two decades. We also identified *int*_SXT_, HS4 and HS5 sequences, belonging to these ICEs, in marine metagenome datasets.

### ICEs characterization

Thirteen multidrug-resistant strains from cholera outbreaks in Nigeria (n = 12, 2009–2010) and a strain from Nepal (n = 1, 1994) were identified harboring ICEs (Table S1). *V. cholerae* O1 strains from Nigeria (n = 12, 2009–2010) and Nepal (n = 1, 1994) showed susceptibility to tetracycline (MIC 1 mg/L) and florfenicol (MIC≤8 mg/L), reduced susceptibility to chloramphenicol (MIC 6–8 mg/L), and resistance to both streptomycin (MIC>1024 mg/L) and trimethoprim/sulfamethoxazole (MIC>32 mg/L), which represents an increased resistance profile compared with early seventh-pandemic isolates and resembles a resistance phenotype encoded by some ICEs, such as the one harbored by epidemic *V. cholerae* O1 lineage from Haiti [8] [11]. These resistant strains carried the *int*_SXT_ gene, indicating the presence of an ICE. Class 1 and class 2 integrons were not found in the strains carrying ICE and mutations in *gyrA* and *parC*, associated with resistance to quinolones, have been previously observed only in the strains from Nigeria [12].

A strain from Nigeria (VC833) and a strain from Nepal (VC504) were selected for ICE characterization by the whole genome sequencing approach. In general the ICEs have been named based on the species and country where they were identified [4] therefore, the ICEs characterized here were named ICE*Vch*Nig1 and ICE*Vch*Nep1. They represent the first ICEs characterized in *V. cholerae* O1 strains from these countries, where cholera has been epidemic and endemic for decades [12] [13].

The ICE*Vch*Nig1 and ICE*Vch*Nep1 contained the same set of 94 open reading frames (ORFs), including the core genes, the five HS and variable regions. These ICEs differed from ICE*Vch*Hai1 by only two (ICE*Vch*Nig1) and eight (ICE*Vch*Nep1) single-nucleotide polymorphism along the entire sequence. Such SNPs occurred in the intergenic regions, except by one that corresponds to a silent mutation in the *pol* gene coding for a DNA polymerase V. Therefore, these polymorphisms did not impact the transmissibility of these ICEs.

**Figure 1:**
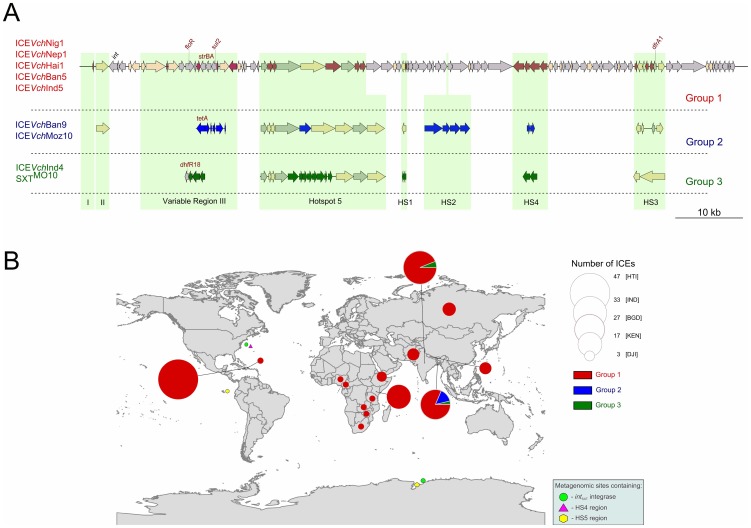
Structure and worldwide distribution of Integrative and Conjugative Elements (ICEs) in epidemic *V. cholerae* strains. (A) The upper line represents the genetic organization of the 2010 Nigeria *V. cholerae* O1 ICE (Group 1). Regions of variability among previously sequenced ICEs (variable regions), and previously identified hotspots of homologous recombination (HS1–HS5) are indicated in light green shading. The core and dispensable genes are indicated by gray and cream arrows, respectively. Unique genes of type 1, 2 and 3 groups are indicated in red, blue and green arrows, respectively. Genes associated with antimicrobial drug resistance *floR* (chloramphenicol), *strAB* (streptomycin), *sul2* (sulfamethoxazole), and *dfrA1*/*dhfR18* (trimethoprim) are written in red. The ICE group 1 have the same structure and gene content in ICE*Vch*Ind5, ICE*Vch*Ban5, ICE*Vch*Nig1 and ICE*Vch*Nep1; similar occurs with group 2 (including ICE*Vch*Ban9 and ICE*Vch*Moz10), and group 3 (including ICE*Vch*Ind4 and SXT^MO10^) have the same gene content and structure. (B) Spatial distribution of ICEs siblings found in epidemic *V. cholerae*. The circles are proportional to abundance found in epidemic *V. cholerae* genomes and the colors are accord to ICE group. The metagenome sites where were obtained BLAST hits are depicted.

Aiming to access the gene content and diversity of the complete ICE sequences, a comparative genomic analysis of ICE*Vch*Nig1 and ICE*Vch*Nep1 with all ICE sequences available (db-mml.sjtu.edu.cn/ICEberg/) was performed. Considering the ICEs structure and gene content, three groups were observed distributed within epidemic *V. cholerae* O1 strains. The ICEs within each of these groups had similar gene content and organization (Figure 1A). There are 58 ICEs described in *V. cholerae* but only eight have a complete sequence available (db-mml.sjtu.edu.cn/ICEberg/). Since ICE*Vch*Ban8 does not encode an *int*_SXT_ orthologous, it is not considered a member of the SXT/R391 family [5] and was not included in our analysis.

Most of the ICEs (ICE*Vch*Nig1, Nep1, Hai1, Ban5 and Ind5) belong to group 1; the group 2 and group3 contain two other ICEs each. These groups are distinguished by different gene content in the variable regions (VR) I, II and III as well as in the HS 1–5 (Figure 1A). The variable region III and HS3 tend to accumulate most of the antimicrobial resistance genes harbored by these ICEs. The ICEs from group 1 contain in the VR-III the *floR*; *strAB*; *sul2* antimicrobial resistance genes. The ICEs from group 2 and group 3 contain this same set of genes in addition to *tet*(A) and *dfrA18*, respectively (Table 1). ICEs from group 1 contain a *dfrA1* gene in the HS3 region (Figure 1A). BLASTN searches were performed against the GenBank and this analysis revealed that the HS4 and HS5 nucleotide region is unique and characteristic of group 1 ICEs. The HS4 and HS5 gene content are represented by four ORFs (∼5.4 kb) and nine ORFs (15 kb), respectively and assigned by RAST [14] and PGAAP [15] mainly as hypothetical proteins. These regions could be used as markers to identify, specifically, ICEs from group 1 distribution.

**Table 1:** Groups of integrative and conjugative elements with similar gene content found in epidemic *V. cholerae* O1.

	ICE name	Host strain	Country and year of isolation	Resistance profile	Accession	References
Group 1						
	ICE*Vch*Nig1	VC833, O1	Nigeria, 2010	*floR, strAB, sul2, dfrA1*	KC886258	This study
	ICE*Vch*Nep1	VC504, O1	Nepal, 1994	*floR, strAB, sul2, dfrA1*	KC886257	This study
	ICE*Vch*Ind5	Ind5, O1	India, 1994	*floR, strAB, sul2, dfrA1*	GQ463142	[5]
	ICE*Vch*Ban5	Ban5, O1	Bangladesh, 1998	*floR, strAB, sul2, dfrA1*	GQ463140	[5]
	ICE*Vch*Hai1	2010EL-1786,O1	Haiti, 2010	*floR, strAB, sul*2, *dfrA1*	JN648379	[8]
Group 2						
	ICE*Vch*Ban9	MJ1236, O1	Bangladesh, 1994	*floR, strAB, sul2, dfrA1* a,*tet*(A)	CP001485	[5]
	ICE*Vch*Moz10	B33, O1	Mozambique, 2004	*floR, strAB, sul2, tet*(A*′*)	ACHZ00000000	[5]
Group 3						
	SXT^MO10^	MO10, O139	India, (1992) 2002	*dfrA18* b, *floR, strAB, sul*2	AY055428	[25]
	ICE*Vch*Ind4	Ind4, O139	India, 1997	*floR, strAB, sul*2	GQ463141	[5]

a *dfrA1* is absent in ICE*Vch*Moz10;

b *dfrA18* is absent in ICE*Vch*Ind4;

NA, not available.

### ICEs distribution

Most studies infer the presence of ICEs just by the antimicrobial resistance profile and identification of the *int*_SXT_ gene [16] [17] [18] [19] without showing the complete ICE gene content, which actually characterizes an ICE. In order to establish the scenario of the distribution of group 1, 2 and 3 ICEs, we performed a screening of 206 *V. cholerae* genomes (GenBank, Mutreja et al [7] and Hendriksen et al [20] using as queries the *int*_SXT_ sequence (marker of SXT/R391 ICEs), and group 1 HS4 and HS5 regions.

We identified 140 *V. cholerae* O1 genomes harboring the entire sequence of group 1 ICE (Table S2). So far, the group 1 ICEs are composed by the siblings: ICE*Vch*Ind5 (India, 1994–2005) [9] [5], ICE*Vch*Ban5 (Bangladesh, 1998) [5], ICE*Vch*Hai1 (Haiti, 2010) [8], ICE*Vch*Nig1 (Nigeria, 2010) and ICE*Vch*Nep1 (Nepal,1994) (this study).

The 140 group 1 ICEs are distributed among strains from India (1984–2009), Bangladesh (1991–2011), Nepal (1994–2010), Zimbabwe (2003), Zambia (2004), Kenya (2005–2009), Russia (2005–2012), Djibouti (2007), Tanzania (2009), South Africa (2009), Pakistan (2009–2010), Cameroon (2010), Nigeria (2010), Haiti (2010–2011) and the Dominican Republic (2011) (Figure 1B, Table S2), showing a widespread distribution as well as their persistence.

Of note, recently, Valia et al, 2013 [21] suggested that siblings of ICE*Vch*Ind5, prevalent in altered *V. cholerae* O1 strains causing epidemics in the Indian subcontinent and Haiti had spread to Africa, once they identified ICE*Vch*Ang3, sibling of ICE*Vch*Ind5, in two *V. cholerae* O1 from Angola (2006) [21]. Herein, the identification of ICE*Vch*Nig1, sibling of ICE*Vch*Ind5, ICE*Vch*Ang3 and ICE*Vch*Hai1, in epidemic *V.* O1 from Nigeria (2009–2010) shows that, in fact, West Africa is resembling the scenario that has been occurring in the Indian subcontinent and in East African countries [9] [22].

One notable factor in the ongoing evolution of pandemic cholera was the acquisition of the SXT/R391 ICE family [7]. Although the genetic relatedness of some of these ICEs were previously inferred [5] [8], our results showed a dispersion of siblings of group 1 ICE over three continents and their persistence for more than two decades. Their persistence and widespread distribution could be due to their presence in a *V. cholerae* O1 lineage and/or because this mobile element can easily be transferred and maintained by positive selection among *V. cholerae* populations. Therefore, we speculate that beyond their multidrug resistance conferred by a set of antimicrobial resistance genes, several hypothetical proteins, encoded in the HS4 and HS5 regions, could have a role in the fitness of the strains.

### Metagenomic analyzes

In order to gain insights into the possible origin of HS4 and HS5 sequences, unique and exclusive regions of siblings of group 1 ICE, we searched for their presence in environmental metagenomic datasets. Hits were obtained for HS4 and HS5 ORFs as well as the *int*_SXT_ gene in the Sargasso sea, a mangrove in the Fernandina Island (Galapagos) and in the Ace Lake Vestfold Hills (Antarctic) (Figure 1B and Table 2). These hits could be due to the presence of epidemic *V. cholerae* O1 harboring this group 1 ICEs. To test this hypothesis, we searched for the presence of the major virulence determinants of epidemic *V. cholerae* O1 (CTXΦ and TCP) in these environmental metagenomic datasets and no hits were obtained. Thus, SXT/R391 ICEs, HS4 and HS5 are present in marine environments away from the direct influence of a cholera pandemic area.

**Table 2:** BLAST search results using HS4 and HS5 ORFs from the group 1 ICE in metagenomic datasets.

Hotspot	ORF	Length (bp)	Function	Metagenomic project	Metagenomic site	No. of hits	Hit size (bp)	Identity (%)	E-value
HS5									
	1	801	hypothetical protein	AAM^a^	Station 365 (Antartic)	1	233	96	10^−100^
	2	549	hypothetical protein	-	-	-	-	-	-
	3	3690	FIG00912888: hypothetical protein	AAM	Station 354 (Antartic)	3	364	96	10^−157^
					Station 365 (Antartic)		348	93	10^−133^
					Station 365 (Antartic)		530		
	4	3741	putative type II restriction enzyme methylase subunit	AAM	Station 236 (Antartic)	2	251	94	10^−102^
					Station 365 (Antartic)		530		
				GOS^b^	Fernandina Island (Galapagos)	1	930	85	0.0
	5	2304	hypothetical protein	GOS	Fernandina Island (Galapagos)	1	996	83	0.0
	6	2043	ATP-dependent protease La	-	-	-	-	-	-
	7	771	hypothetical protein	-	-	-	-	-	-
	8	816	hypothetical protein	-	-	-	-	-	-
HS4									
	1	1617	ATPase involved in DNA repair	-	-	-	-	-	-
	2	762	Transposase ISTB	GOS	Sargasso Station 11	1	756	97	0.0
	3	1455	Transposase IstA	GOS	Sargasso Station 11	1	1017	99	0.0
	4	1080	ATPase involved in DNA repair	-	-	-	-	-	-

a AAM, Antatic Aquatic Metagenome.

b GOS, Global Ocean Sampling Expedition.

## Conclusions

In the present study, the complete sequences of ICEs from Nigeria and Nepal were determined and compared to other complete ICEs from *V. cholerae* O1 strains. We identified three types of ICEs within epidemic *V. cholerae* O1, which had a similar gene content. In the further analysis, performing BLASTn searches against *V. cholerae* O1 genomes, we found that a type of ICEs showed widespread distribution and persistence over two decades.

There is a spatio-temporal prevalence of ICE in *V. cholerae* O1, carrying a set of antimicrobial resistance determinants and exclusive gene content. This element had already been identified and addressed in *V. cholerae* strains from four countries but our study revealed its presence in strains from more ten countries. The prevalence and dispersion of this ICE can be indicative of its role in the fitness of the current pandemic lineage. Moreover, this study revealed the scenario showing the occurrence of this ICE since the current *V. cholerae* ICE nomenclature is based on their geographic origin not considering their gene content. Therefore, these sibling ICEs are in epidemic *V. cholerae* O1, lasting for more than two decades and spreading worldwide.

## Materials and Methods

Forty-one clinical *V. cholerae* O1 strains from the Bacteria Culture Collection of Environment and Health at the Oswaldo Cruz Foundation, FIOCRUZ were included in this study. These strains were storage in MicroBank beads (ProLab Diagnostics) at −70°C. All of them were screened for the presence of genetic elements associated with antimicrobial resistance, such as ICEs, class 1 and class 2 integrons [12].

Susceptibility testing was performed by the disc diffusion method according to the CLSI guidelines [23] using the following commercial antibiotics: erythromycin (15 µg), gentamicin (10 µg), amikacin (30 µg), sulfonamide (300 µg), sulfamethoxazole (25 µg), trimethoprim/sulfamethoxazole (30 µg), trimethoprim (5 µg), chloramphenicol (30 µg), tetracycline (30 µg), ampicillin (10 µg), cefoxitin (30 µg), cefuroxime (30 µg), ceftazidime (30 µg), streptomycin (10 µg), spectinomycin (100 µg), azithromycin (15 µg), nalidixic acid (30 µg), ciprofloxacin (5 µg). The MICs of tetracycline, chloramphenicol, streptomycin and trimethoprim/sulfamethoxazole were also determined by using E-test Strips (BioMerieux). The MIC of florfenicol (Sigma) was addressed by using the agar dilution method [23] on Mueller-Hinton plates containing varying concentrations of this antibiotic ranging from 4 mg/L to 32 mg/L. In this test, the inoculum was brought to an optical density of 1.0 at a wavelength of 610 nm (∼1×10^8^ bacterial cells/ml) according to the McFarland scale. When no interpretive criteria for *V. cholerae* were available based on CLSI guidelines, breakpoints for enterobacteriaceae were applied.

The ICEs gene content and insertion site on the chromosome were determined based on the whole-genome sequencing of VC833 (Nigeria, 2009) and VC504 (Nepal, 1994) strains. Single-end 454 pyrosequencing (GS-Junior from Roche Diagnostics, USA) from the high-throughput platform of Oswaldo Cruz Institute/FIOCRUZ. The reads were assembled by using Newbler (Roche Diagnostics) and UGENE v1.9.8 [24] software. Custom primer walking by using Sanger sequencing closed the gaps of the ICE region. The recovered ICEs were annotated by RAST [14] and PGAAP software [15]. The nucleotide sequences of the identified ICEs were deposited in GenBank under accession numbers KC886258 and KC886257.

Complete ICE sequences were retrieved from the ICEberg database (db-mml.sjtu.edu.cn/ICEberg/), a web-based resource for integrative and conjugative elements found in bacteria. In the ICEberg site, there are 58 *V. cholerae* ICEs described, but only eight of these have entire ICE sequences. So, these eight ICE sequences were used for comparison with the ICE*Vch*Nig1 and ICE*Vch*Nep1. The ICE distribution was addressed using HS4, HS5 and *int*_SXT_ as queries in BLASTN searches against *V. cholerae* genomes from Genbank, Sequence Reads Archive (SRA) [20] and European Nucleotide Archive (ENA) [7]. Moreover, we performed searches against CAMERA metagenome database (camera.calit2.net/) with a similarity threshold based on an e-value below to 1e-20 (April 2014).

## Supporting Information

Table S1 ***V. cholerae*** **O1 strains from the Bacteria Culture Collection of Environment and Health at the Oswaldo Cruz Foundation, FIOCRUZ screened in this study.**
(DOC)

Table S2 ***V. cholerae*** **O1 genomes harboring the group 1 ICE.**
(DOC)
