# Supplementary material for: Worldwide Occurrence of Integrative Conjugative Element Encoding Multidrug Resistance Determinants in Epidemic Vibrio cholerae O1
Source: PLoS One. 2014 Sep 29;9(9):e108728. doi: 10.1371/journal.pone.0108728 (PMC4181655; doi:10.1371/journal.pone.0108728)
Supplement: Table S1 — V. cholerae O1 strains from the Bacteria Culture Collection of Environment and Health at the Oswaldo Cruz Foundation, FIOCRUZ screened in this study. (DOC) [file pone.0108728.s001.doc]

**Table S1.** *V. cholerae* O1 strains from the Bacteria Culture Collection of Environment and Health at the Oswaldo Cruz Foundation, FIOCRUZ screened in this study.

| Strain^a^ | Country | Isolation year | Antimicrobial resistance profile | int_SXT_/class 2 integron |
| --- | --- | --- | --- | --- |
| VC33 | Tanzania | 1979 | Susceptible | -/- |
| VC34 | Ghana | 1970 | Susceptible | -/- |
| VC37 | Ghana | 1980 | Susceptible | -/- |
| VC79 | Nigeria | 1971 | STP | -/- |
| VC92 | Ghana | 1980 | STP, SPT | -/+ |
| VC94 | Ghana | 1979 | STP, SPT | -/+ |
| VC95 | Ghana | 1978 | STP, SPT | -/+ |
| VC96 | Ghana | 1978 | STP, SXT | -/- |
| VC97 | Ghana | 1979 | STP, SPT | -/+ |
| VC98 | Ghana | 1979 | STP, SPT | -/+ |
| VC102 | Ghana | 1979 | STP | -/- |
| VC104 | Algeria | 1972 | STP | -/- |
| VC106 | Ghana | 1980 | Susceptible | -/- |
| VC107 | Ghana | 1980 | Susceptible | -/- |
| VC111 | Nigeria | 1972 | Susceptible | -/- |
| VC121 | India | 1973 | Susceptible | -/- |
| VC192 | Ghana | 1980 | Susceptible | -/- |
| VC193 | Ghana | 1981 | Susceptible | -/- |
| VC225 | Ghana | 1976 | Susceptible | -/- |
| VC869 | Nigeria | 1971 | TRM | -/- |
| VC79 | Nigeria | 1971 | Susceptible | -/- |
| VC111 | Nigeria | 1972 | Susceptible | -/- |
| VC869 | Nigeria | 1971 | SPT | -/- |
| VC504 | Nepal | 1994 | STP, FOX, CXM, AMP, NAL, CIP, SUL, SMZ, SXT, TRM, CHL | +/- |
| VC832 | Nigeria | 2009 | STP, CXM, AMP, NAL, CIP, SUL, SMZ, SXT, TRM, CHL | +/- |
| VC841 | Nigeria | 2009 | STP, AMP, NAL, CIP, SUL, SMZ, SXT, TRM, CHL | +/- |
| VC833 | Nigeria | 2010 | STP, FOX, CXM, AMP, NAL, CIP, SUL, SMZ, SXT, TRM, CHL | +/- |
| VC834 | Nigeria | 2010 | AMK , STP, FOX, AMP, NAL, CIP, SUL, SMZ, SXT, TRM, CHL | +/- |
| VC835 | Nigeria | 2010 | STP, FOX, AMP, NAL, CIP, SUL, SMZ, SXT, TRM, CHL | +/- |
| VC991 | Nigeria | 2010 | STP, FOX, AMP, NAL, CIP, SUL, SMZ, SXT, TRM, CHL | +/- |
| VC997 | Nigeria | 2010 | STP, FOX, AMP, NAL, CIP, SUL, SMZ, SXT, TRM, CHL | +/- |
| VC1001 | Nigeria | 2010 | STP, FOX, NAL, CIP, SUL, SMZ, SXT, TRM, CHL | +/- |
| VC1007 | Nigeria | 2010 | STP, FOX, AMP, NAL, CIP, SUL, SMZ, SXT, TRM, CHL | +/- |
| VC836 | Nigeria | 2010 | STP, AMP, NAL, CIP, SUL, SMZ, SXT, TRM, CHL | +/- |
| VC996 | Nigeria | 2010 | AMP, SUL, SMZ, SXT, TRM | -/- |
| VC998 | Nigeria | 2010 | SUL, SMZ, SXT, TRM | -/- |
| VC999 | Nigeria | 2010 | STP, NAL, CIP, SUL, SMZ, SXT, TRM, CHL | +/- |
| VC1004 | Nigeria | 2010 | AMK, STP, CAZ, NAL, CIP, SUL, SMZ, SXT, TRM, CHL | +/- |
| VC1006 | Nigeria | 2010 | SUL, SMZ, SXT, TRM | -/- |
| VC1009 | Nigeria | 2010 | NAL, SUL, SMZ, SXT, TRM | -/- |
| VC1005 | Nigeria | 2010 | AMP, NAL, SUL, SMZ, SXT, TRM | -/- |

^a^ All strains were recovered from human feces, except by the environmental VC1005 strain isolated from river water.

Abbreviations: STP, streptomycin; SPT, spectinomycin; SXT, trimethoprim/sulfamethozaxole; TRM, trimethoprim; CAZ, ceftazidime; FOX, cefotin; CXM, cefuroxime; AMP, ampicillin; NAL, nalidixic acid; CIP, ciprofloxacin; SUL, sulfonamide; SMZ, sulfamethoxazole; CHL, chloramphenicol; AMK, amikacin. The disc diffusion method was applied and interpreted according to CLSI guidelines [23].
