# Supplementary material for: Worldwide Occurrence of Integrative Conjugative Element Encoding Multidrug Resistance Determinants in Epidemic Vibrio cholerae O1
Source: PLoS One. 2014 Sep 29;9(9):e108728. doi: 10.1371/journal.pone.0108728 (PMC4181655; doi:10.1371/journal.pone.0108728)
Supplement: Table S2 — V. cholerae O1 genomes harboring the group 1 ICE. (DOC) [file pone.0108728.s002.doc]

Table S2. *V. cholerae* genomes harboring the group 1 ICE.

| **Strain name^a^** | **Isolation place** | **Isolation year** | **Accession Number** |
| --- | --- | --- | --- |
| PRL64 | India | 1984 | ERS013139 |
| V5 | India | 1989 | ERS013130 |
| A130 | India | 1989 | ERS013177 |
| A131 | India | 1989 | ERS013178 |
| V109 | India | 1990 | ERS013131 |
| MG116025 | Bangladesh | 1991 | ERS013135 |
| MG116226 | Bangladesh | 1991 | ERS013282 |
| VC073 | Bangladesh | 1994 | ERS013262 |
| VC504 | Nepal | 1994 | KC886257 |
| AR-32732 | Bangladesh | 1999 | ERS013260 |
| IB4662 | Bangladesh | 2001 | ERS013238 |
| E1978 | Bangladesh | 2001 | ERS013259 |
| MQ1273 | Bangladesh | 2001 | ERS013261 |
| MQ4 | Bangladesh | 2001 | ERS013263 |
| CIRS101 | Bangladesh | 2002 | ACVW01000003 |
| Nep-21113 | Nepal | 2003 | APGF01000022 |
| CP1038 | Zimbabwe | 2003 | ALDC01000006 |
| Nep-21106 | Nepal | 2003 | APGE01000085 |
| CP1041 | Zambia | 2004 | ALDE01000018 |
| MBRN14 | India | 2004 | ERS013127 |
| MBN17 | India | 2004 | ERS013134 |
| IB4322 | India | 2004 | ERS013254 |
| IB4339 | India | 2004 | ERS013270 |
| RND18826 | Russia | 2005 | AYOM01000194 |
| 3500-05 | India | 2005 | AHGL01000025 |
| 6191 | Kenya | 2005 | ERS013209 |
| 6215 | Kenya | 2005 | ERS013211 |
| 6193 | Kenya | 2005 | ERS013212 |
| 6195 | Kenya | 2005 | ERS013214 |
| 6196 | Kenya | 2005 | ERS013215 |
| IB4519 | India | 2005 | ERS013239 |
| RND18899 | Russia | 2006 | AYNN01000130 |
| 3546-06 | India | 2006 | AHGM01000066 |
| A488 | Bangladesh | 2006 | ERS013129 |
| IB4642 | India | 2006 | ERS013255 |
| IB4656 | India | 2006 | ERS013258 |
| A488 | Bangladesh | 2006 | ERS013279 |
| A483 | Djibouti | 2007 | ERS013198 |
| A487 | Bangladesh | 2007 | ERS013199 |
| A481 | Djibouti | 2007 | ERS013206 |
| A482 | Djibouti | 2007 | ERS013207 |
| 6180 | Kenya | 2007 | ERS013208 |
| 6214 | Kenya | 2007 | ERS013210 |
| 6194 | Kenya | 2007 | ERS013213 |
| 6197 | Kenya | 2007 | ERS013216 |
| 6201 | Kenya | 2007 | ERS013217 |
| 6210 | Kenya | 2007 | ERS013218 |
| 6212 | Kenya | 2007 | ERS013219 |
| IB4646 | India | 2007 | ERS013237 |
| IB4538 | India | 2007 | ERS013269 |
| A487 | Bangladesh | 2007 | ERS013281 |
| IB4593 | India | 2007 | ERS013268 |
| IB4605 | India | 2007 | ERS013257 |
| IB4552 | India | 2007 | ERS013233 |
| IB4551 | India | 2007 | ERS013266 |
| IB4623 | India | 2007 | ERS013267 |
| IB4536 | India | 2007 | ERS013240 |
| IB4600 | India | 2007 | ERS013236 |
| IB4585 | India | 2007 | ERS013232 |
| IB4122 | India | 2007 | ERS013264 |
| IB4488 | India | 2007 | ERS013234 |
| IB4593 | India | 2007 | ERS013268 |
| 3554-08 | Nepal | 2008 | AHGN01000015 |
| 2009V-1046 | Pakistan | 2009 | AHGP01000001 |
| 2011EL-1137 | South Africa | 2009 | AHGJ01000010 |
| 2009V-1116 | Pakistan | 2009 | AHGA01000074 |
| 2009V-1096 | India | 2009 | AHFZ01000016 |
| 2009V-1085 | India | 2009 | AHFY01000072 |
| 2009V-1046 | Pakistan | 2009 | AHFX01000069 |
| 2009V-1131 | India | 2009 | AHGB01000062 |
| IDHO1_726 | India | 2009 | ERS013147 |
| 7682 | Kenya | 2009 | ERS013220 |
| 7684 | Kenya | 2009 | ERS013221 |
| 7685 | Kenya | 2009 | ERS013224 |
| 7686 | Kenya | 2009 | ERS013225 |
| 7687 | Kenya | 2009 | ERS013226 |
| IB4784 | Tanzania | 2009 | ERS013235 |
| VC833 | Nigeria | 2010 | KC886258 |
| RND19187 | Russia | 2010 | AYNM01000153 |
| PCS023 | Bangladesh | 2010 | APGG01000027 |
| NHCC-010F | Bangladesh | 2010 | APGD01000027 |
| NHCC-006C | Bangladesh | 2010 | APGB01000028 |
| NHCC-004A | Bangladesh | 2010 | APGA01000073 |
| EM-1546 | Bangladesh | 2010 | APFW01000034 |
| EDC-020 | Bangladesh | 2010 | APFT01000033 |
| EC-0012 | Bangladesh | 2010 | APFQ01000022 |
| EC-0009 | Haiti | 2010 | APFP01000081 |
| HC-81A2 | Haiti | 2010 | ALEA01000128 |
| HC-56A2 | Haiti | 2010 | ALDX01000142 |
| HC-46A1 | Haiti | 2010 | ALDQ01000012 |
| HC - 42A1 | Haiti | 2010 | ALDO01000118 |
| HC-39A1 | Haiti | 2010 | ALDM01000121 |
| HC-20A2 | Haiti | 2010 | ALDL01000011 |
| CP1048 | Bangladesh | 2010 | ALDJ01000007 |
| H1 | Haiti | 2010 | AKGH01000001 |
| HC-69A1 | Haiti | 2010 | AJSP01000026 |
| HC-62B1 | Haiti | 2010 | AJSO01000082 |
| HC-37A1 | Haiti | 2010 | AJSJ01000107 |
| HC-17A2 | Haiti | 2010 | AJSI01000096 |
| HC-77A1 | Haiti | 2010 | AJRW01000091 |
| HC-62A1 | Haiti | 2010 | AJRV01000027 |
| 2011EL-1089 | Haiti | 2010 | AHGH01000012 |
| 2010V-1014 | Pakistan | 2010 | AHGG01000066 |
| 2010EL-2010N | Haiti | 2010 | AHGF01000010 |
| 2010EL-2010H | Haiti | 2010 | AHGE01000011 |
| 2010EL-1961 | Haiti | 2010 | AHGD01000010 |
| 2010EL-1749 | Cameroon | 2010 | AHGC01000016 |
| HC-81A1 | Haiti | 2010 | AGVC02000005 |
| HC-80a1 | Haiti | 2010 | AGVB02000100 |
| HC-7A1 | Haiti | 2010 | AGVA02000009 |
| HC-72A2 | Haiti | 2010 | AGUY02000084 |
| HC-71A1 | Haiti | 2010 | AGUX02000027 |
| HC-68A1 | Haiti | 2010 | AGUW02000030 |
| HC-67A1 | Haiti | 2010 | AGUV02000028 |
| HC-65A1 | Haiti | 2010 | AGUU02000033 |
| HC-64A1 | Haiti | 2010 | AGUT02000021 |
| HC-61A1 | Haiti | 2010 | AGUS01000007 |
| HC-43A1 | Haiti | 2010 | AGUQ01000014 |
| HC-33A2 | Haiti | 2010 | AGUP01000028 |
| HC-32A1 | Haiti | 2010 | AGUO01000022 |
| HC-28A1 | Haiti | 2010 | AGUN01000093 |
| HC-23A1 | Haiti | 2010 | AGUM01000004 |
| HC-22A1 | Haiti | 2010 | AGUL01000089 |
| HC-21A1 | Haiti | 2010 | AGUK01000001 |
| HC-19A1 | Haiti | 2010 | AGUJ01000034 |
| HC-06A1 | Haiti | 2010 | AGUI01000094 |
| HC-38A1 | Haiti | 2010 | AFOV01000052 |
| HC-70A1 | Haiti | 2010 | AFON01000119 |
| HC-49A2 | Haiti | 2010 | AFOM01000012 |
| 2010EL-1792 | Haiti | 2010 | AELJ01000054 |
| 2010EL-1798 | Haiti | 2010 | AELI01000092 |
| HC-17A1 | Haiti | 2010 | AJRN01000395 |
| HC-40A1 | Haiti | 2010 | AFOK01000144 |
| HC-48A1 | Haiti | 2010 | AFOL01000151 |
| HC-48B2 | Haiti | 2010 | AGUR01000108 |
| EC-0027 | Bangladesh | 2011 | APFR01000085 |
| 2011EL-301 | Russia | 2011 | AJFN02000021 |
| 2011V-1021 | Dominican Republic | 2011 | AHGK01000009 |
| 2011EL-1133 | Haiti | 2011 | AHGI01000002 |
| RND6878 | Russia | 2012 | AYNL01000104 |

^a^ All strains correspond to clinical samples.
